# Assessing the Reliability of Hip Physical Examination via Telemedicine: Intra- and Inter-observer Analysis in Osteoarthritis, Total Hip Arthroplasty, and Normal Hips

**DOI:** 10.7759/cureus.85369

**Published:** 2025-06-04

**Authors:** Fabio Seiji M Yamaguchi, Lucas S Bombonato, Bruno A Rudelli, Helder S Miyahara, Henrique Melo de Campos Gurgel

**Affiliations:** 1 Hip Surgery Department of the Institute of Orthopedics and Traumatology (IOT), Clinics Hospital of School of Medicine of the University of Sao Paulo, Sao Paulo, BRA

**Keywords:** hip examination, hip joint, orthopaedic practices, telemedicine in orthopedics, total hip arthroplasty (tha)

## Abstract

Background

Telemedicine is an emerging tool in orthopedic care, offering remote assessments for patients with limited mobility or in underserved areas. However, its use for hip joint exams remains understudied. This study addresses that gap by evaluating intra- and inter-observer reliability of telemedicine-based hip exams in osteoarthritis, total hip arthroplasty, and asymptomatic cases. We analyzed 100 hips from 50 patients, treating each hip independently. Notably, this is the first study to incorporate a standardized instructional video to enhance examiner consistency.

Objective

This study aimed to evaluate the reliability of hip joint physical examinations performed via telemedicine compared to traditional in-person evaluations in individuals with hip osteoarthritis, total hip arthroplasty (THA), and normal hips. We hypothesized that telemedicine-based assessments would demonstrate substantial intra- and inter-observer reliability, supporting their potential integration into orthopedic clinical practice. The parameters assessed included lower limb muscle trophism, claudicating gait, the Trendelenburg test, hip range of motion (flexion, extension, abduction, adduction, internal and external rotation), and the Timed Up and Go (TUG) test.

Methods

This was a cross-sectional, analytical, and comparative study involving 100 hips from 50 patients. Each patient contributed with both hips, which were evaluated individually. Participants were categorized into three groups based on the clinical condition of each hip: hip osteoarthritis (n=33), THA (n=33), and normal hips (n=34). Thus, a single patient could have hips classified into different groups. Each hip was treated as an independent observational unit to enable a detailed biomechanical and functional analysis across the groups. However, for the Timed Up and Go (TUG) test, since it is a global functional assessment, the evaluation was performed per patient rather than per hip. Unlike the other tests, which focused on hip-specific mobility, the TUG test reflects overall patient function. Therefore, the sample size for this test was 50.

Results

Hip flexion, extension, abduction, adduction, internal and external rotation, and the Timed Up and Go (TUG) test showed good to excellent reliability (kappa > 0.6; Intraclass Correlation Coefficient (ICC) > 0.99). Claudicating gait demonstrated moderate to very good agreement, varying by clinical group. In contrast, muscle trophism and the Trendelenburg test showed low reliability (kappa < 0.6), possibly due to inadequate lighting, camera positioning, or difficulty distinguishing subtle visual cues. Notably, the use of a standardized instructional video prior to the virtual exam contributed to higher reliability when compared to previously published studies. No adverse events or complications occurred in patients with total hip arthroplasty or osteoarthritis during the remote assessments, reinforcing the safety of the method.

Conclusion

Telemedicine proved to be a reliable and safe method for assessing most functional aspects of the hip, particularly joint range of motion and gait. The use of a pre-recorded instructional video enhanced the consistency of virtual assessments, supporting its integration into routine orthopedic practice, especially when in-person evaluation is not feasible.

## Introduction

Telemedicine has become an increasingly important tool in modern healthcare, particularly in improving access to care in underserved or geographically isolated areas. Its origins date back to the Middle Ages, when physicians would exchange correspondence to guide remote treatments during epidemics [1,2]. Over time, technological advancements such as the telegraph, telephone, and, more recently, video conferencing have enabled remote medical practice to become a practical reality. The COVID-19 pandemic further accelerated the adoption of telemedicine across specialties, including orthopedics, where it has proven valuable for postoperative care and rehabilitation [3].

Despite these advances, telemedicine in orthopedic physical examinations, especially of complex joints like the hip, remains underexplored. Hip assessments typically require palpation, evaluation of range of motion, gait observation, and orthopedic testing, which are fundamental for diagnosing conditions such as hip osteoarthritis (OA), also referred to as coxarthrosis, and evaluating post-operative function in total hip arthroplasty (THA) [4,5]. As highlighted in a recent systematic review, while telemedicine has been increasingly validated for joints such as the knee and shoulder, there remains a significant lack of robust evidence supporting its reliability in hip assessments, underscoring the need for further research in this area [6].

This study addresses this gap by evaluating both intra- and inter-observer reliability of hip physical examination via telemedicine in three distinct clinical scenarios: OA, THA, and asymptomatic (normal) hips. A robust sample of 100 hips from 50 patients was analyzed, with each hip treated as an independent observational unit. Notably, this is the first study to incorporate a standardized instructional video prior to telemedicine assessments, aiming to enhance examiner consistency. In addition, we evaluate the reliability of the Timed Up and Go (TUG) test - a key functional metric in hip evaluation - when performed remotely. By establishing and validating a reproducible methodology, this research contributes to the standardization of telemedicine protocols in orthopedic clinical practice.

## Materials and methods

Ethical approval

This study was approved by the Research Ethics Committee of Hospital of the Clinics of the Faculty of Medicine of the University of São Paulo, under approval number: 5.153.695, dated December 8, 2021. All participants provided written informed consent prior to enrollment.

Study objective

This study aimed to evaluate the reliability of hip joint physical examinations performed via telemedicine compared to traditional in-person evaluations in individuals with hip osteoarthritis (OA), total hip arthroplasty (THA), and normal hips. We hypothesized that telemedicine-based assessments would demonstrate substantial intra- and inter-observer reliability, supporting their potential integration into orthopedic clinical practice. The parameters assessed included lower limb muscle trophism, claudicating gait, the Trendelenburg test, hip range of motion (flexion, extension, abduction, adduction, internal and external rotation), and the Timed Up and Go (TUG) test.

Study design and sample

This was a cross-sectional, analytical, and comparative study involving 100 hips from 50 patients. Each patient contributed with both hips, which were evaluated individually. Participants were categorized into three groups based on the clinical condition of each hip: hip osteoarthritis (n=33), THA (n=33), and normal hips (n=34). Thus, a single patient could have hips classified into different groups. Each hip was treated as an independent observational unit to enable a detailed biomechanical and functional analysis across the groups [7-9].

However, for the Timed Up and Go (TUG) test, since it is a global functional assessment, the evaluation was performed per patient rather than per hip. Unlike the other tests, which focused on hip-specific mobility, the TUG test reflects overall patient function. Therefore, the sample size for this test was 50.

Sample size calculation

Sample size was calculated using G*Power software (Heinrich Heine University, Dusseldorf, Germany). A moderate effect size (0.7) was adopted as per Cohen (1988), with a statistical power of 80%, to ensure feasibility and statistical robustness while minimizing Type II errors [10-13].

Inclusion and exclusion criteria

Inclusion Criteria

Individuals aged 18 years or older with (1) clinically and radiographically confirmed hip OA, (2) a postoperative THA period greater than three months, or (3) no hip pathology confirmed by clinical and imaging evaluation were included.

Exclusion Criteria

The exclusion criteria were (1) other hip conditions unrelated to OA, (2) patients confined to wheelchairs or bedridden, (3) individuals with partial hip arthroplasty or spacer placement, (4) severe musculoskeletal deformities or major motor deficits, and (5) cognitive impairments limiting test execution.

Assessment procedures

In-Person Evaluation

**Figure 1:**
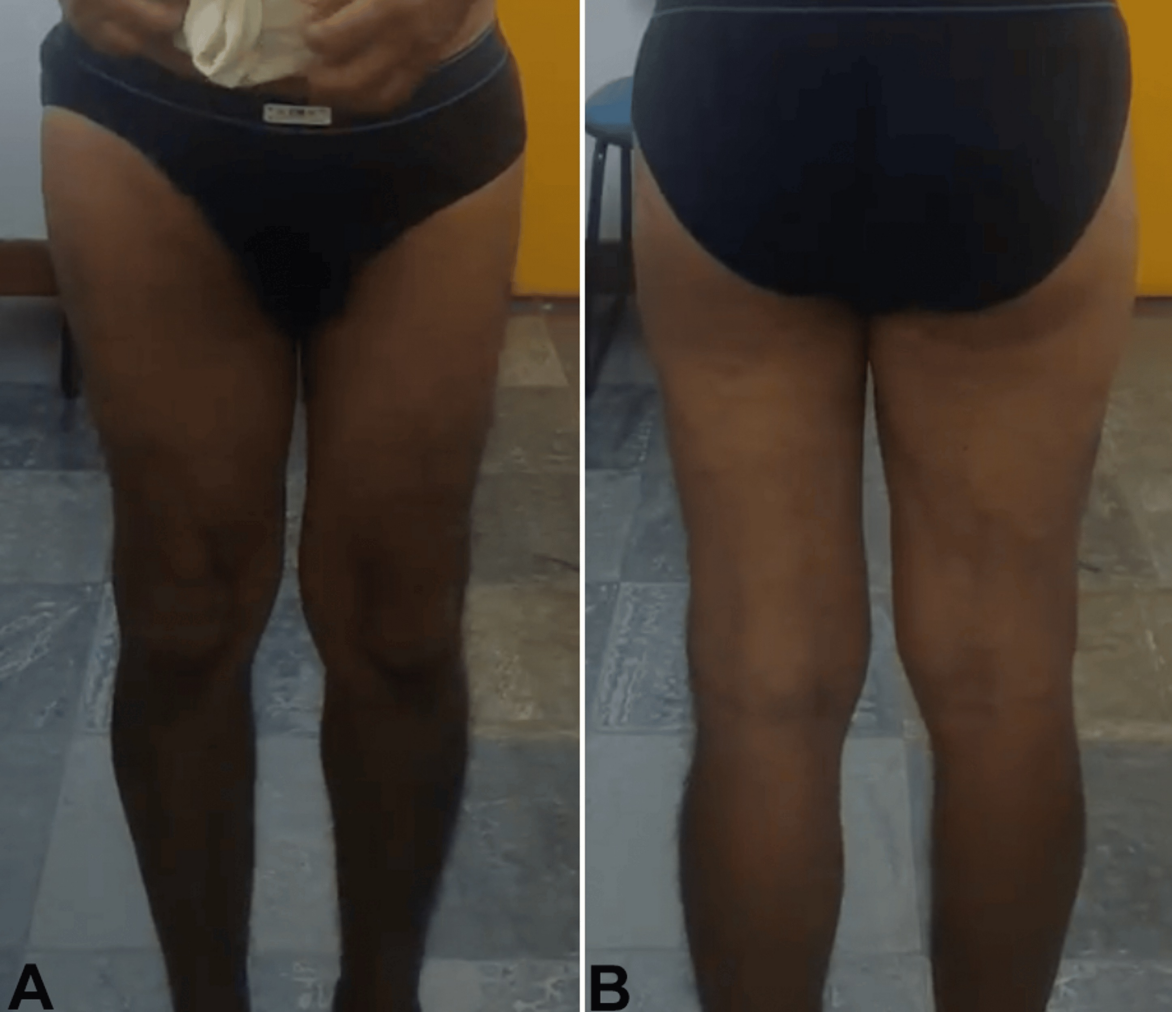
Muscle trophism: posterior (A) and anterior (B) evaluation of the lower limbs.

**Figure 2:**
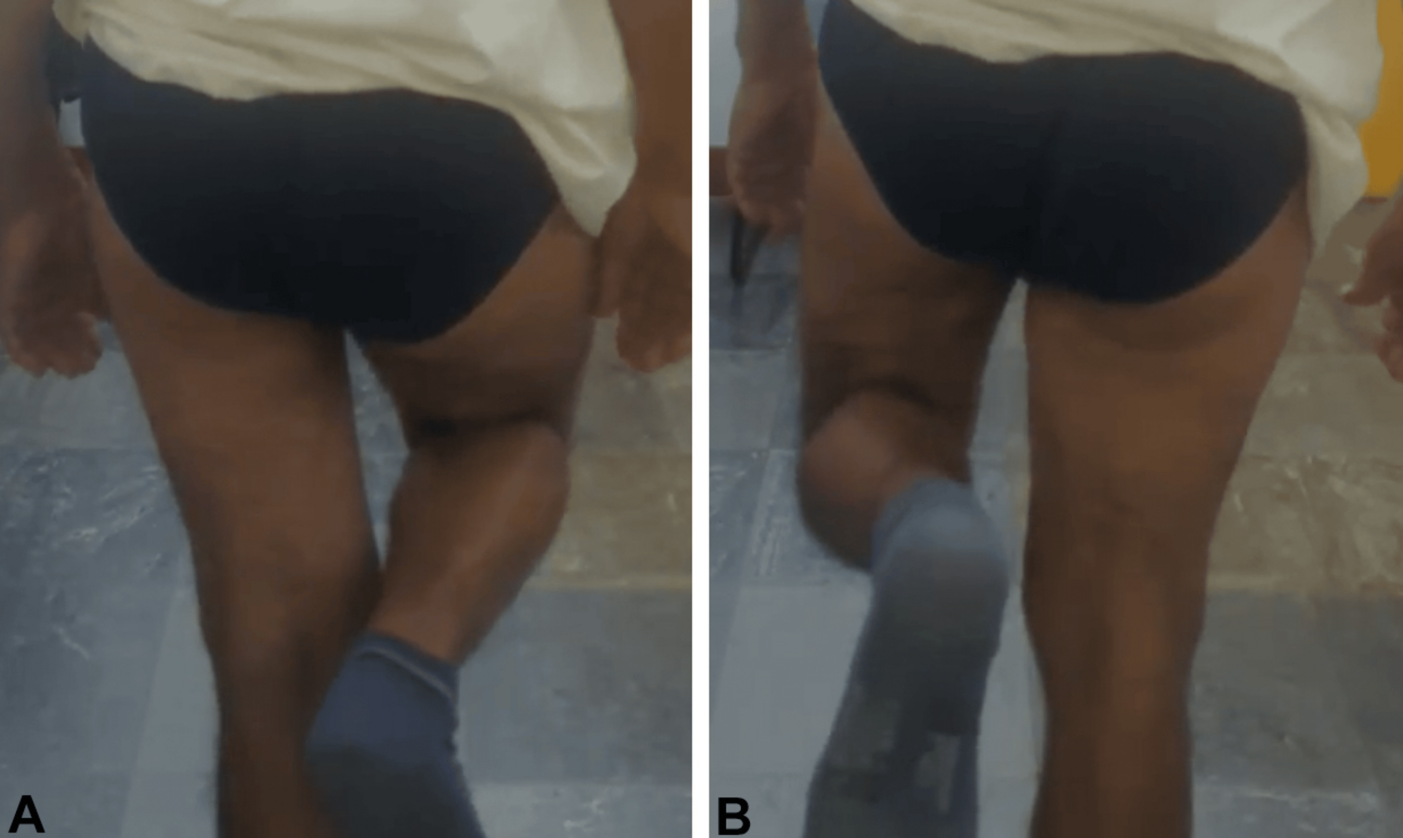
Trendelenburg test on the left (A) and on the right (B)

**Figure 3:**
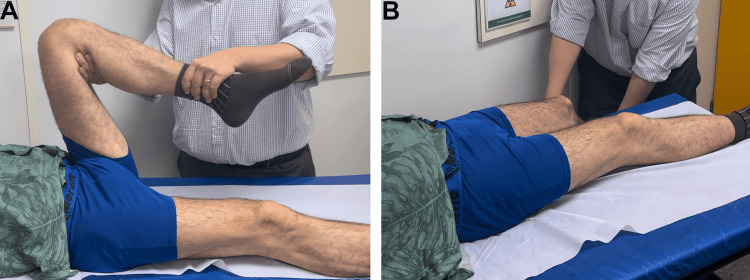
Flexion (A) and extension (B) of the left hip

**Figure 4:**
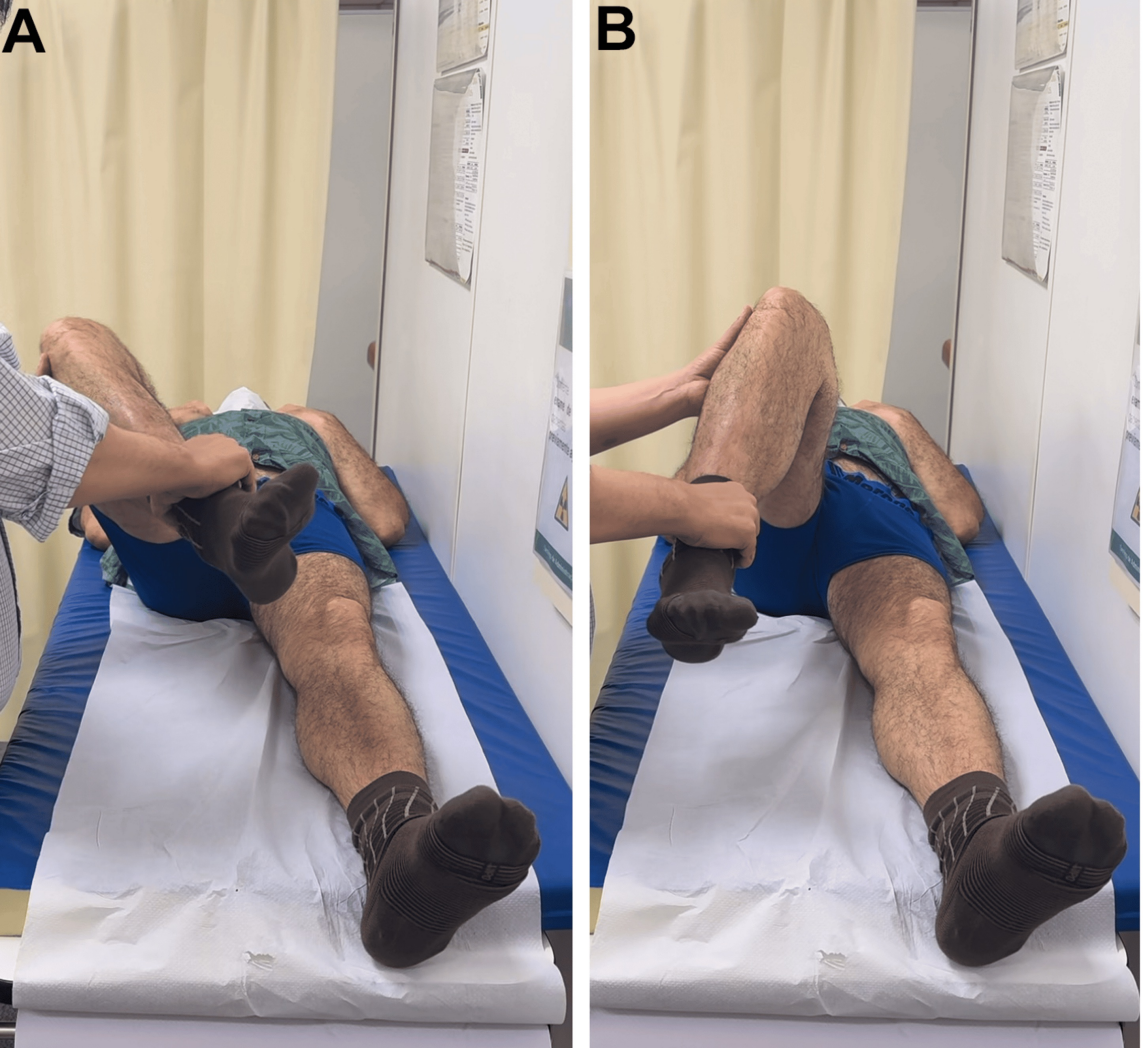
External rotation (A) and internal rotation (B) of the right hip

**Figure 5:**
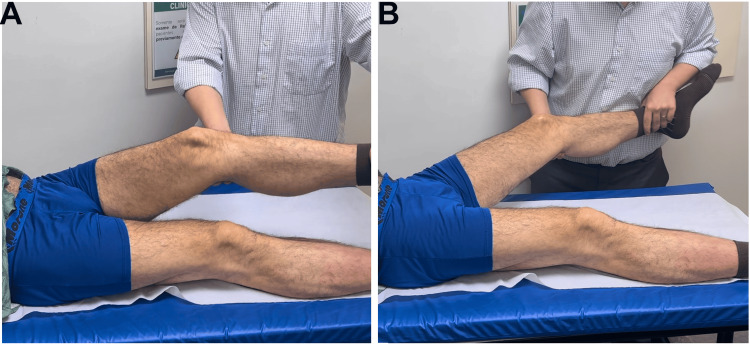
Adduction (A) and abduction (B) of the left hip

All participants underwent a standardized physical examination conducted by a hip orthopedic surgeon. The assessment protocol included evaluation of lower limb muscle trophism, claudicating gait, Trendelenburg sign, and range of motion (flexion, extension, abduction, adduction, internal and external rotation). Functional capacity was assessed via the TUG test (Figures 1-5).

Telemedicine Evaluation

Following the in-person assessment, patients watched a standardized instructional video presented before the virtual physical examination. The video demonstrated step-by-step instructions for each movement and test to ensure consistency and reproducibility during the remote assessment (Video 1).

**Video 1:** Standardized instructional video Standardized instructional video presented to patients before the virtual physical examination. The video demonstrated step-by-step instructions for each movement and test to ensure consistency and reproducibility during the remote assessment.

**Figure 6:**
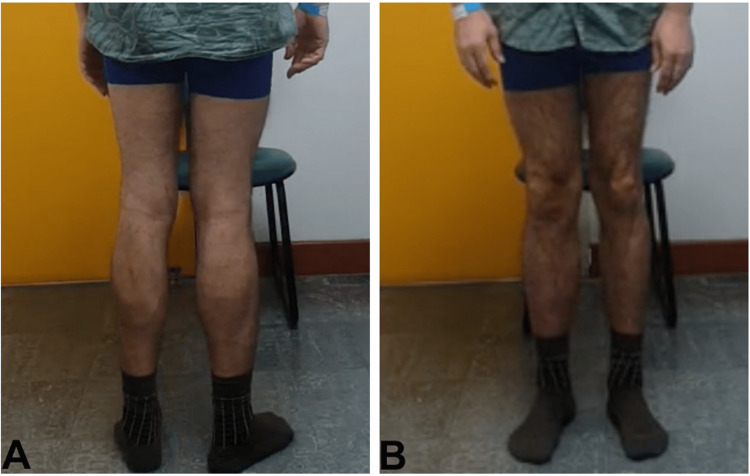
Muscle trophism: posterior (A) and anterior (B) evaluation of the lower limbs

**Figure 7:**
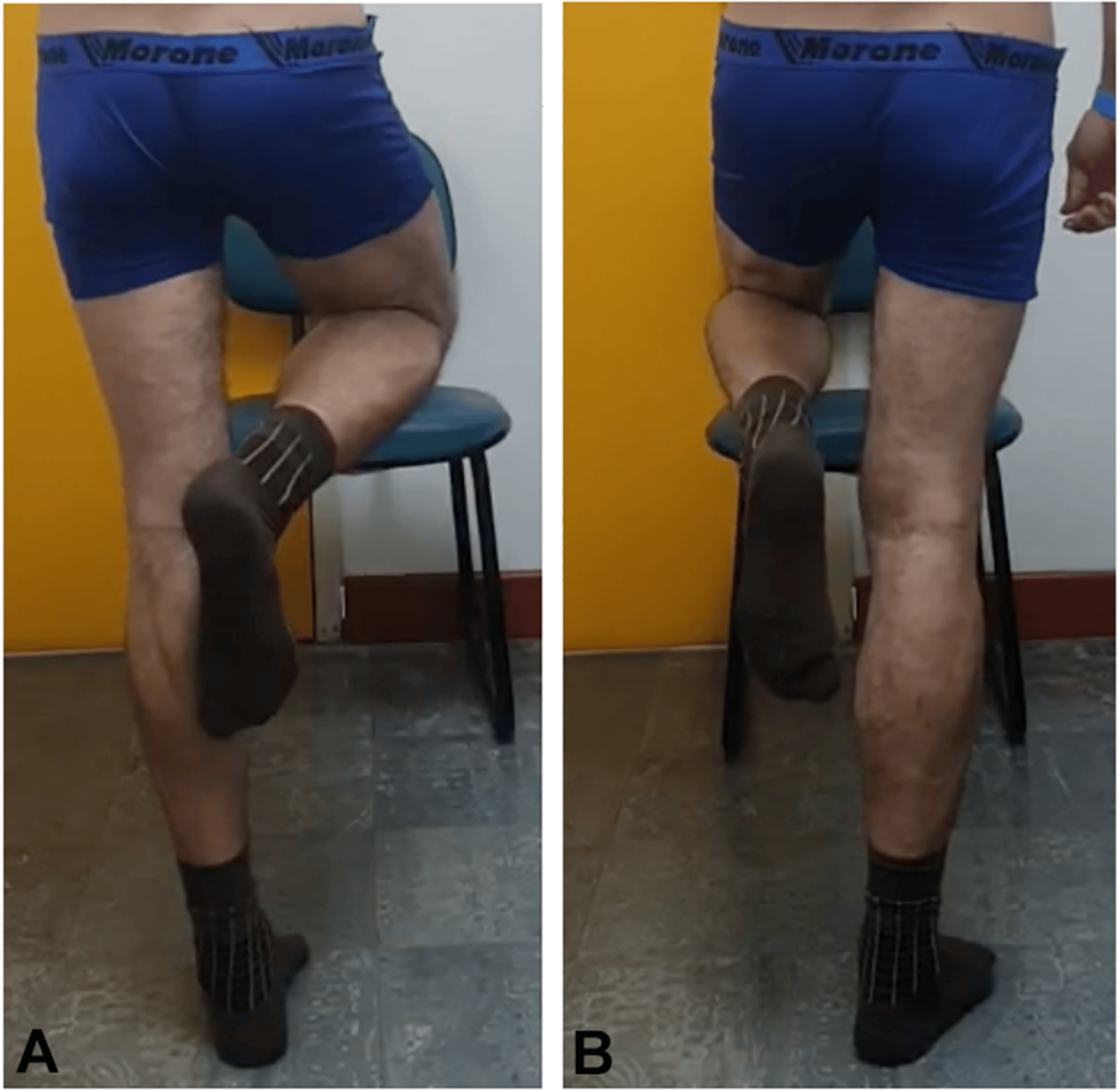
Trendelenburg test on the left (A) and on the right (B)

**Figure 8:**
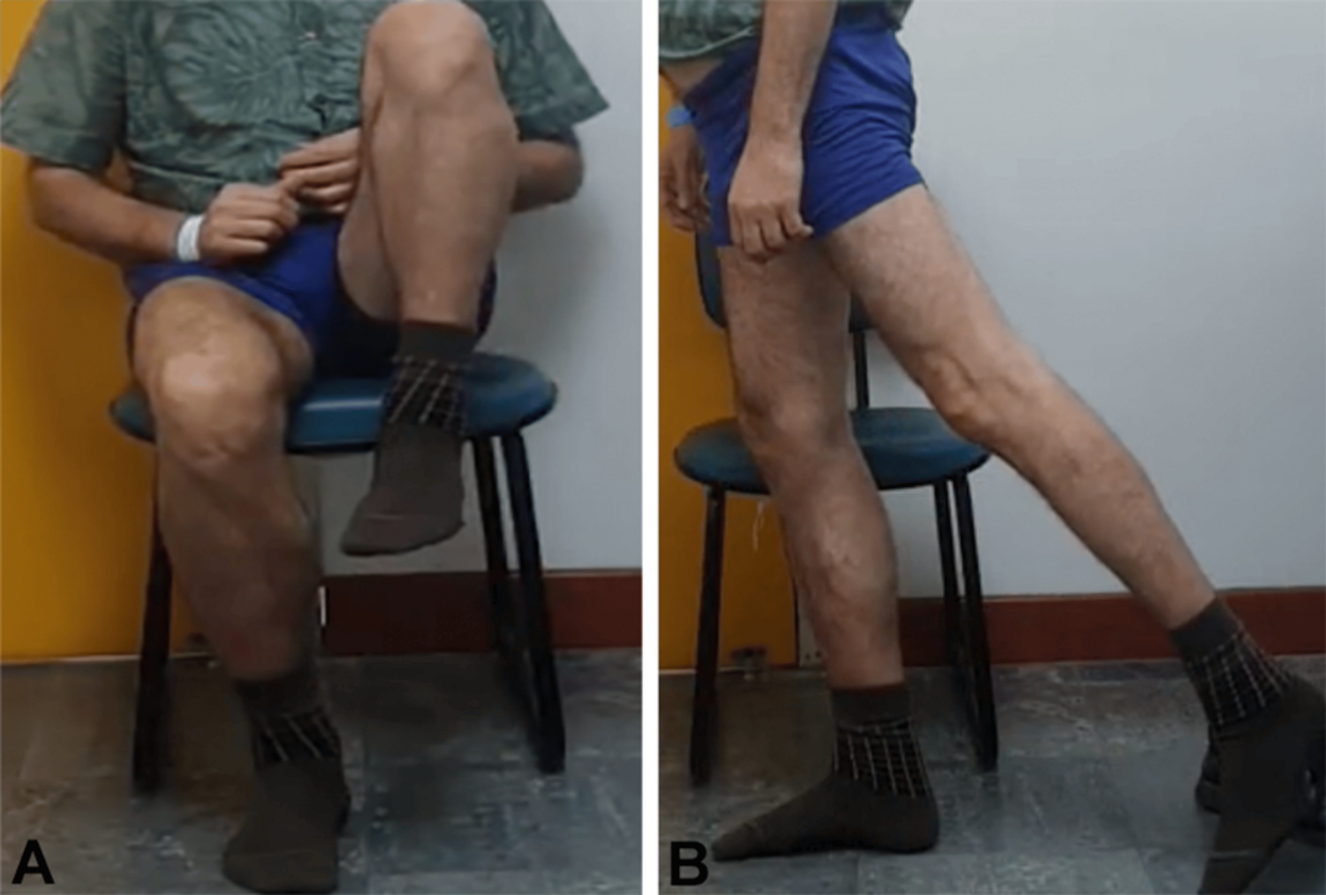
Flexion (A) and extension (B) of the left hip

**Figure 9:**
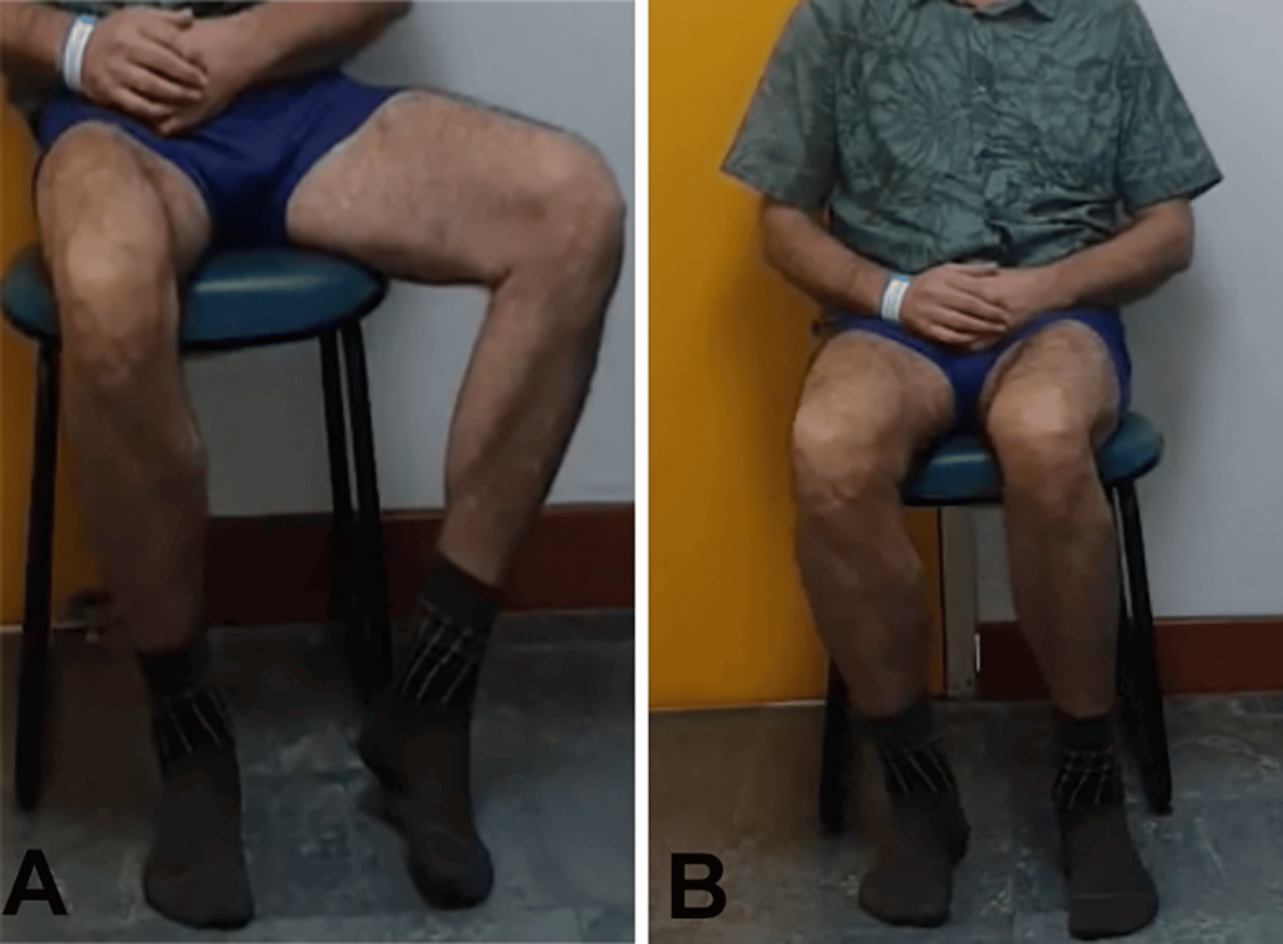
Abduction (A) and adduction (B) of the left hip

**Figure 10:**
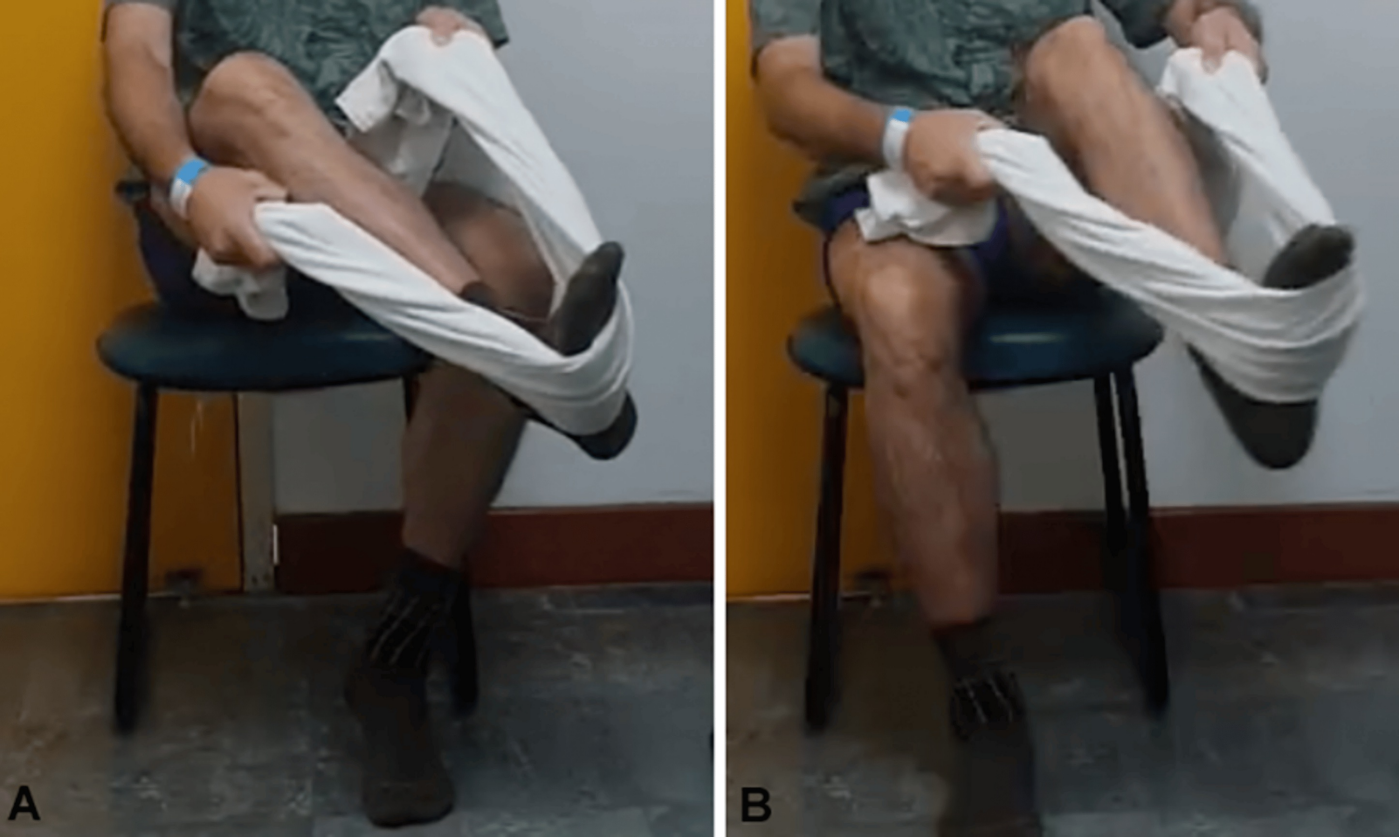
External rotation of the right hip (A) and internal rotation of the left hip (B)

The remote physical examination was then conducted and video-recorded for subsequent analysis. The same parameters assessed in-person were evaluated during the virtual examination (Figures 6-10).

Reliability assessment

Intra-observer Reliability

To reduce recall bias, Observer 1 re-evaluated all video recordings six months after the original in-person assessment. Video sequences were presented in random order.

Inter-observer Reliability

A second hip orthopedic surgeon, blinded to previous assessments, independently reviewed the video recordings to determine inter-observer agreement.

Statistical analysis

Agreement between in-person and virtual assessments was analyzed using raw agreement percentages and Cohen’s Kappa coefficient (κ) with 95% confidence intervals. Kappa values were interpreted as follows: ≤0.20 poor, 0.21 to 0.40 fair, 0.41 to 0.60 moderate, 0.61 to 0.80 good, 0.81 to 0.99 very good, and 1.00 perfect agreement [14]. Kappa values above 0.6 were considered acceptable for orthopedic clinical practice.

For the TUG test, quantitative agreement was evaluated using the Intraclass Correlation Coefficient (ICC), Bland-Altman plots, Pearson’s correlation coefficient (r), and scatter plots. ICC values were interpreted as: <0.40 poor, 0.40 to 0.59 fair, 0.60 to 0.74 good, and ≥0.75 very good or excellent [15]. Paired t-tests were applied to compare TUG values across in-person and remote conditions. All statistical analyses were performed using STATA/MP 18.0 (StataCorp, College Station, USA) and MedCalc 23.1.6 (Medcalc Software Ltd., Ostend, Belgium), with significance set at p < 0.05.

Equipment

Remote evaluations were conducted using a Samsung Galaxy S10 Plus (Samsung Electronics, Suwon, South Korea) to ensure high-resolution imaging. TUG test timing was measured with a digital stopwatch along a 3-meter marked pathway.

## Results

In orthopedic practice, Kappa values above 0.6 (good) indicate strong agreement and are widely accepted as evidence of the reliability of clinical assessment methods. Therefore, a Kappa coefficient exceeding this threshold demonstrates that telemedicine is a reliable tool for clinical evaluations.

No adverse events occurred in patients with osteoarthritis or THA, reinforcing the safety of remote assessment.

Table 1 summarizes the interpretation of Kappa coefficient (κ) values in relation to the corresponding degree of agreement.

**Table 1:** Degree of agreement

Kappa's value	Degree of agreement
0.00 - 0.20	Poor
0.21 - 0.40	Reasonable
0.41 - 0.60	Moderate
0.61 - 0.80	Good
0.81 - 0.99	Very good
1.0	Perfect

Muscle trophism assessment in lower limbs: moderate concordance in in-person evaluations and good agreement in video comparisons

The assessment of lower limb muscle trophism concordance revealed that in the hip osteoarthritis and THA groups, comparisons between in-person and video examinations showed reasonable to moderate agreement (κ = 0.248 to 0.426 for hip osteoarthritis and κ = 0.372 to 0.426 for THA), while video-based assessments by both observers demonstrated good agreement (κ = 0.779 for hip osteoarthritis and κ = 0.791 for THA), indicating higher consistency when using the same method. In the normal hip group, in-person versus video assessments showed moderate agreement (κ = 0.471, moderate), whereas video-to-video evaluations exhibited very good concordance (κ = 0.872, very good). These findings suggest that video-based methods consistently yield more reliable results, especially when applied by both observers, across the studied groups (Tables 2, 3, 4).

**Table 2:** Summary of kappa measures for each assessment - hip osteoarthritis

Assessment Method		Muscle Trophism	Claudicating Gait	Trendelenburg	Hip Flexion	Extension	Abduction	Adduction	Internal Rotation	External Rotation
In-person	Vídeo_Obs1_	Reasonable	Moderate	Reasonable	Good	Very good	Very good	Very good	Good	Very good
In-person	Vídeo_Obs2_	Reasonable	Moderate	Poor	Good	Very good	Very good	Very good	Good	Good
Vídeo_Obs1_	Vídeo_Obs2_	Good	Very Good	Reasonable	Good	Very good	Very good	Very good	Good	Very good

**Table 3:** Summary of kappa measures for each assessment - THA THA: total hip arthroplasty

Assessment Method		Muscle Trophism	Claudicating Gait	Trendelenburg	Hip Flexion	Extension	Abduction	Adduction	Internal Rotation	External Rotation
In-person	Vídeo_Obs1_	Reasonable	Very good	Moderate	Good	Very good	Perfect	Very good	Very good	Good
In-person	Vídeo_Obs2_	Moderate	Perfect	Moderate	Good	Good	Perfect	Good	Very good	Good
Vídeo_Obs1_	Vídeo_Obs2_	Good	Very good	Moderate	Good	Good	Perfect	Very good	Very good	Good

**Table 4:** Summary of kappa measures for each assessment - normal

Assessment Method		Muscle Trophism	Claudicating Gait	Trendelenburg	Hip Flexion	Extension	Abduction	Adduction	Internal Rotation	External Rotation
In-person	Vídeo_Obs1_	Moderate	Good	Moderate	Perfect	Perfect	Good	Perfect	Very good	Perfect
In-person	Vídeo_Obs2_	Reasonable	Very good	Reasonable	Perfect	Perfect	Good	Good	Very good	Perfect
Vídeo_Obs1_	Vídeo_Obs2_	Very good	Very good	Good	Perfect	Perfect	Perfect	Good	Very good	Perfect

Claudicating gait assessment: high concordance in THA and normal hip, moderate agreement in hip osteoarthritis

The assessment of claudicating gait concordance revealed that in the hip osteoarthritis group, the highest agreement was observed between video evaluations by both observers, with a raw agreement of 97.0% and a kappa coefficient of 0.872 (very good), while in-person and video comparisons showed moderate concordance (κ = 0.522 and 0.436). In the THA group, the highest concordance occurred between video evaluations (κ = 0.937, very good) and between in-person and video assessments by Observer 2 (κ = 1.000, perfect). In the normal hip group, the best agreement was also found between video evaluations (κ = 0.866, very good), while in-person versus video assessments by Observer 1 and 2 showed good (κ = 0.721) and very good (κ = 0.850) concordance, respectively (Table 2, 3, 4).

Trendelenburg test assessment: moderate concordance in THA and normal hip, poor to fair agreement in hip osteoarthritis

The evaluation of Trendelenburg test concordance revealed that in the hip osteoarthritis group, the highest agreement was observed between in-person and video assessments by Observer 1 (78.8%, κ = 0.374, fair), while the comparison with Observer 2 showed poorer agreement (69.7%, κ = 0.167). Video evaluations between both observers also demonstrated fair agreement (72.8%, κ = 0.372). In the THA group, moderate agreement was noted, with the best concordance observed between in-person and video evaluations by Observer 2 (75.8%, κ = 0.517), and video evaluations between observers also showing moderate concordance (72.7%, κ = 0.449). In the normal hip group, video evaluations between both observers presented the highest agreement (88.2%, κ = 0.640, good), while in-person versus video assessments by Observer 1 and 2 showed moderate (κ = 0.573) and fair (κ = 0.402) concordance, respectively (Table 2, 3, 4).

Hip flexion assessment: good concordance in hip osteoarthritis and THA, perfect agreement in normal hip

The assessment of hip flexion concordance in the hip osteoarthritis group showed good agreement between in-person and video evaluations by both observers, with raw agreements of 87.8% (κ = 0.727) and 84.8% (κ = 0.651), respectively, while the video-to-video comparison showed higher concordance (90.9%, κ = 0.791). In the THA group, the best agreement was observed between in-person and video evaluations by Observer 1 (90.9%, κ = 0.809, very good), followed by Observer 2 (87.9%, κ = 0.742, good), and video evaluations between both observers also presented good agreement (90.9%, κ = 0.791). In the normal hip group, all comparisons demonstrated perfect concordance, with a raw agreement of 100% and a kappa coefficient of 1.000 (Tables 2, 3, 4).

Hip extension assessment: very good concordance in hip osteoarthritis and THA, perfect agreement in normal hip

The assessment of hip extension in the hip osteoarthritis group showed very good agreement between in-person and video evaluations, with Observer 1's kappa coefficient of 0.835 and Observer 2's κ of 0.820. Both observers also showed very good agreement when video evaluations were compared. In the THA group, Observer 1 had a very good agreement (κ = 0.872), while Observer 2's agreement was good (κ = 0.615). Video-to-video evaluations in this group showed good agreement (κ = 0.716). In the normal hip group, all evaluations showed perfect concordance, with a kappa coefficient of 1.000 (Tables 2, 3, 4).

Hip abduction assessment: very good concordance in hip osteoarthritis, perfect agreement in THA, and good to perfect agreement in normal hip

The assessment of hip extension concordance in the hip osteoarthritis group showed very good agreement between in-person and video evaluations, with raw agreements of 94.0% (κ = 0.835) for Observer 1 and 93.9% (κ = 0.820) for Observer 2. The video-to-video evaluations between both observers also showed very good concordance (93.9%, κ = 0.820). In the THA group, the best agreement was observed between in-person and video assessments by Observer 1 (97.0%, κ = 0.872), followed by Observer 2 (90.9%, κ = 0.615, good). The comparison between video evaluations by both observers showed good agreement (93.9%, κ = 0.716). In the normal hip group, all comparisons demonstrated perfect concordance, with a raw agreement of 100% and a kappa coefficient of 1.000 (Tables 2, 3, 4).

High concordance in hip adduction assessment across hip osteoarthritis, THA, and normal hip groups 

The hip adduction evaluation in the hip osteoarthritis group showed high agreement between in-person and video assessments. The concordance between Observer 1 and video assessments was very good, with a raw agreement of 96.9% and a kappa coefficient (κ) of 0.936. The comparison with Observer 2 also showed very good agreement, with a raw agreement of 93.9% and κ = 0.874. Between the two video observers, the agreement remained very good (97.0%, κ = 0.937). In the total hip arthroplasty (THA) group, the best agreement was observed between in-person and video assessments by Observer 1 (97.0%, κ = 0.841), followed by Observer 2 (93.9%, κ = 0.716, good). The comparison between video observers showed very good agreement (97.0%, κ = 0.841). In the normal hip group, perfect agreement was found between in-person and video assessments (100%, κ = 1.000), while video-to-video comparisons also showed good agreement (κ = 0.653) (Tables 2, 3, 4).

Hip internal rotation assessment: good concordance in hip osteoarthritis, very good agreement in THA and normal hip 

The assessment of hip internal rotation in the hip osteoarthritis group showed good agreement between in-person and video evaluations, with a raw agreement of 94.0% and a kappa coefficient (κ) of 0.764. Video evaluations by both observers also exhibited good agreement, with the same raw agreement and kappa coefficient. In the THA group, the highest agreement was found between video evaluations by both observers, showing a raw agreement of 94.0% and a kappa coefficient (κ) of 0.876, classified as very good. In-person and video assessments by Observer 1 also demonstrated very good agreement (90.9%, κ = 0.812). In the normal hip group, both in-person and video assessments showed very good agreement (97.1%, κ = 0.892), as did the video-to-video evaluations (97.1%, κ = 0.892) (Tables 2, 3, 4).

High concordance in external hip rotation assessment for normal and THA groups, with moderate results in hip osteoarthritis

The assessment of external rotation in the hip osteoarthritis group showed high agreement between in-person and video evaluations. The raw agreement between in-person and video assessments by Observer 1 was 97.0% with a kappa coefficient (κ) of 0.891, indicating very good concordance. The agreement between Observer 2's in-person and video evaluations was slightly lower, with a raw agreement of 94.0% and a kappa coefficient (κ) of 0.796, classified as good. Both video assessments exhibited very good concordance (97.0%, κ = 0.891). External hip rotation in the THA group showed slightly lower agreement compared to the osteoarthritis group, despite the THA group having higher concordance in other variables. Crude agreement between video and in-person assessments was high (0.970) for both Observer 1 and Observer 2. However, kappa coefficients varied: 0.653 for Observer 1 and 0.784 for Observer 2, both indicating good agreement. Inter-observer video analysis showed a crude agreement of 0.941 and a kappa of 0.784. External rotation was more difficult to assess in the THA group due to prosthetic mechanical limitations, protective patient behavior, and reduced movement variability. These factors may lead to less pronounced, more homogeneous movements, lowering the kappa despite high crude agreement. In contrast, the normal hip group showed perfect agreement (κ = 1.000) in all comparisons, demonstrating that in the absence of joint abnormalities, external rotation can be reliably assessed via telemedicine (Tables 2, 3, 4).

High reliability and slight overestimation of TUG test measurements using video assessment: clinically irrelevant differences

**Figure 11:**
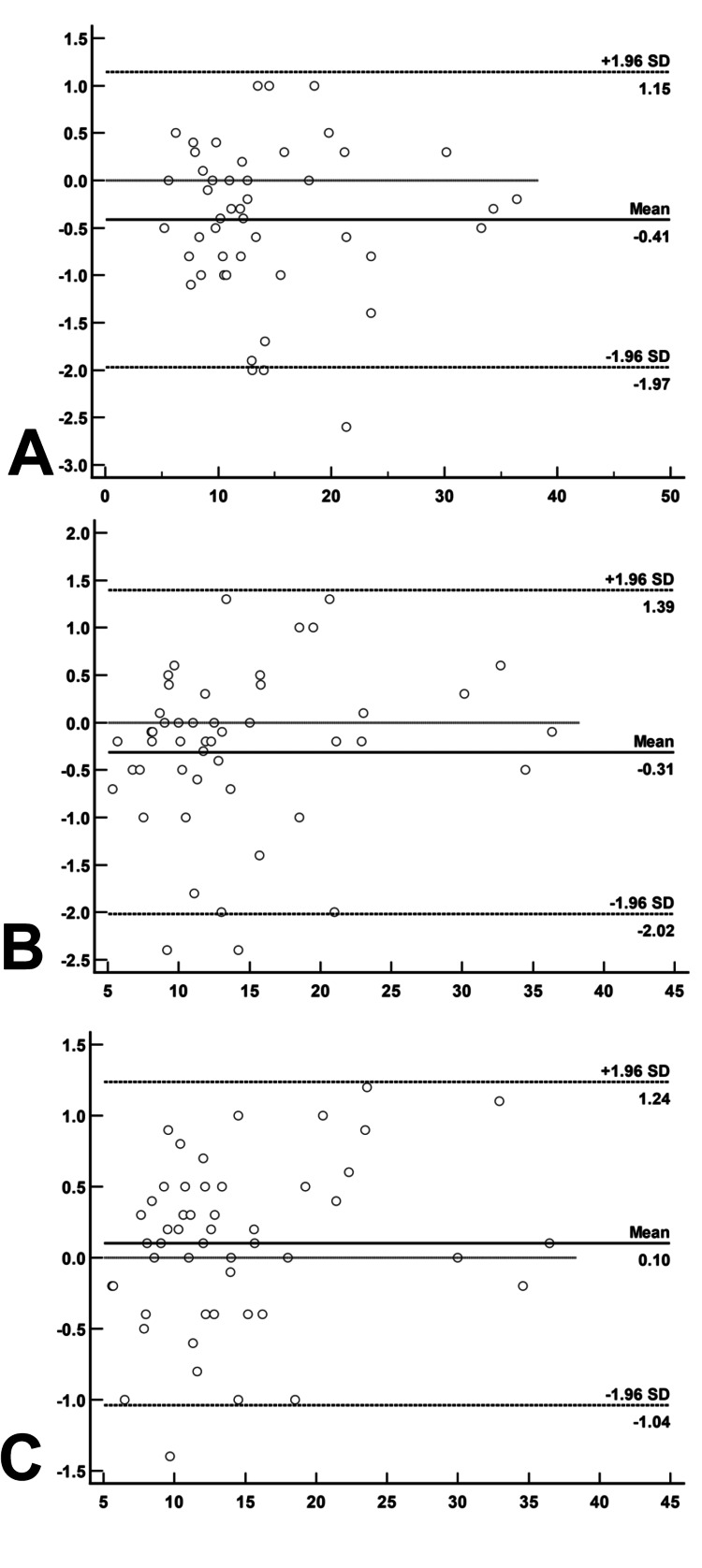
Bland-Altman plot for Timed Up and Go (TUG) test performed in-person and via video by both observers for study participants Plot A: Mean between in-person TUG and video_Obs1 x Difference between in-person TUG and video_Obs 1 Plot B: Mean between TUG video_Obs1 and video_Obs 2 x Difference between TUG video_Obs1  and video_Obs 2 Plot C:Mean between TUG video_Obs1 and video_Obs 2 x Difference between TUG video_Obs1  and video_Obs 2

The TUG test results demonstrated a high level of reliability between in-person and video-based assessments, with ICCs ranging from 0.992 to 0.997. The Bland-Altman (Figure 11) plots revealed that video measurements were slightly higher than in-person ones, with mean differences of -0.41 ± 0.11 seconds (p<0.001) and -0.31 ± 0.12 seconds (p = 0.015) for comparisons between in-person and video Observer 1, and in-person and video Observer 2, respectively. Despite the observed difference, the variation between the two video observers was not statistically significant (p = 0.229).

**Figure 12:**
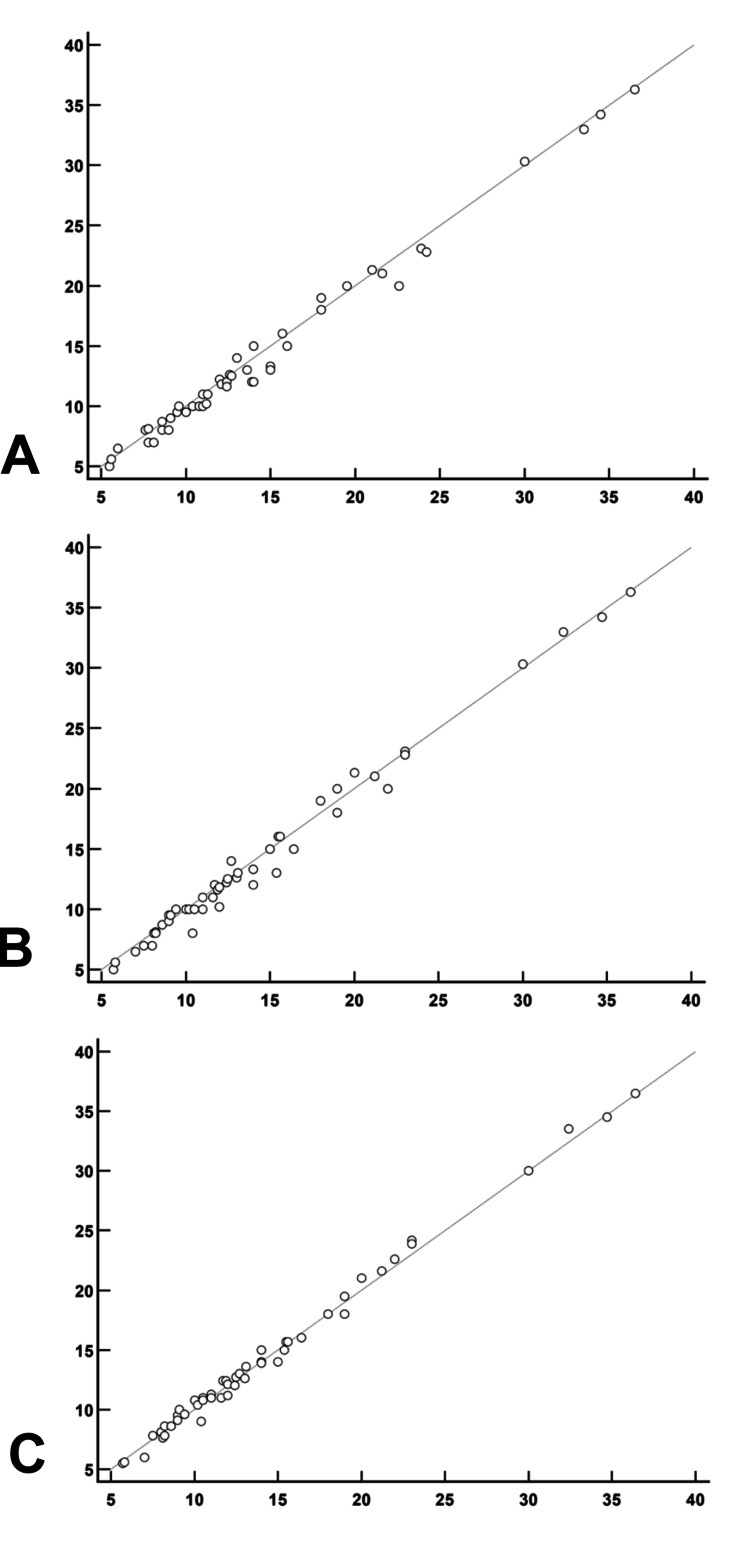
Scatter plot for Timed Up and Go (TUG) test performed in-person and via video by both observers for study participants Plot A: TUG test video_Obs 1 X  In-person TUG test Plot B: TUG test video_Obs 2 X  In-person TUG test Plot C: TUG test video_Obs 2 X  TUG test video_Obs 1

Additionally, Pearson's correlation indicated a strong linear relationship between the methods, with an alignment close to 45°, indicating high concordance (Figure 12).

The comparison between in-person and video TUG tests showed a higher concentration of points with negative differences, suggesting that video measurements consistently exceeded in-person values. The difference between video Observer 1 and Observer 2 was minimal (0.10 ± 0.08 seconds) and not statistically significant. Overall, the high ICC values and the strong linear correlation support the feasibility of using video-based methods for TUG testing.

## Discussion

This study is the first to assess the reliability of physical examination of the hip joint via telemedicine using 100 hips from 50 patients, thereby enhancing statistical power while reflecting realistic clinical scenarios where bilateral pathology is common. This distinction is important, as earlier studies, such as Jaenisch et al. (2021), which included only 29 patients, relied on smaller cohorts and lacked the statistical robustness needed for broader generalization of findings [16].

As highlighted in a recent systematic review, while telemedicine has been increasingly validated for joints such as the knee and shoulder, there remains a significant lack of robust evidence supporting its reliability in hip assessments, underscoring the need for further research in this area [6].

A key innovation of this study is the individualized assessment of hip movements rather than grouping them into antagonistic pairs (e.g., flexion/extension) [16], a practice observed in previous studies. This granularity increased the precision and interpretability of remote evaluations. Most parameters, including flexion, extension, abduction, adduction, internal and external rotation, and the TUG test, demonstrated good to excellent intra-observer reliability, and strong inter-observer agreement was also observed, indicating the reproducibility of telemedicine protocols for physical examination.

Another major methodological strength was the use of a standardized instructional video, viewed by patients prior to the remote examination. This standardized guidance likely contributed to greater consistency in test execution and scoring, as evidenced by improved agreement metrics when compared with studies that did not implement such tools (e.g., Jaenisch et al., 2021) [16]. Moreover, by making the video publicly accessible through a platform such as YouTube, the study promoted patient autonomy and accessibility, facilitating health literacy. This is a relevant and often underdiscussed factor in telemedicine assessments, particularly in populations with varying educational backgrounds.

However, certain assessments, particularly muscle trophism and the Trendelenburg test, yielded lower inter-observer concordance. These limitations appear to stem not from patient-related variability but rather from inherent challenges in remote evaluation, such as lighting, camera positioning, and absence of palpation. Still, even for these more complex assessments, agreement remained moderate, suggesting that standardization and training can mitigate many of the challenges associated with virtual physical exams [17-19].

Additionally, this study is the first to validate the TUG test for remote use. High intraclass correlation coefficients confirmed its reliability for functional assessment via telemedicine. Moreover, our evaluation of claudicating gait adds a novel dimension to remote orthopedic assessments. Prior literature has rarely differentiated pathological from physiological gait in virtual settings; our results show this is both feasible and clinically informative [20, 21].

Importantly, no adverse events were reported, even among patients with advanced OA and THA, confirming the safety of remote physical examinations. While promising, certain statistical considerations must be acknowledged. The inclusion of bilateral hips may introduce correlation within patients, potentially inflating agreement measures. Furthermore, implementation in routine practice may require adaptation of workflows and infrastructure (e.g., ensuring video quality and patient tech literacy).

In summary, this study not only fills a critical gap in the validation of telemedicine for hip-specific evaluations but also demonstrates that, with appropriate standardization and training, remote physical exams can be reliable, safe, and clinically meaningful tools in orthopedic care.

Study limitations

This study has limitations, including the subjectivity of video-based assessments, which rely solely on visual interpretation and may lead to inter-observer variability. Technical factors such as camera angle, lighting, and video quality can affect standardization. Use of a single in-person examiner may reduce generalizability. Including more diverse participants and multiple evaluators in future studies could enhance methodological robustness.

## Conclusions

This study demonstrated that telemedicine is a reliable and feasible approach for conducting physical examinations of the hip joint, particularly in assessing joint range of motion (flexion, extension, abduction, adduction, internal and external rotation) and functional mobility through the TUG test. These parameters showed good to excellent inter- and intra-observer agreement, supporting their applicability in remote orthopedic evaluations. While claudicating gait assessments also demonstrated moderate to very good reliability, especially in patients with total hip arthroplasty and normal hips, results varied with clinical presentation. In contrast, muscle trophism and the Trendelenburg test showed lower levels of reliability, suggesting that these assessments may be limited in virtual formats due to the need for tactile feedback and visual precision. Notably, no adverse events were reported among patients with hip prostheses or osteoarthritis during remote evaluations, reinforcing the safety of this modality. These findings support the integration of telemedicine into clinical practice for the functional evaluation of the hip, particularly when standardized instructional tools are employed to enhance assessment accuracy.
